# Case Report: Colon malignant tumor caused by retroperitoneal small round cell undifferentiated sarcoma

**DOI:** 10.3389/fonc.2023.1212475

**Published:** 2023-12-21

**Authors:** Yuqin Wei, Zhiyong Zhang, Chenyan Long, Xiaoliang Huang, Weizhong Tang, Xianwei Mo, Jungang Liu

**Affiliations:** ^1^ Guangxi Medical University, Nanning, China; ^2^ Department of General Surgery, Zhuzhou Central Hospital, Zhuzhou, China; ^3^ Division of Colorectal & Anal Surgery, Department of Gastrointestinal Surgery, Guangxi Medical University Cancer Hospital, Nanning, China

**Keywords:** small round cell undifferentiated sarcoma, colon malignant tumor, retroperitoneal, diagnosis, therapy, case report

## Abstract

Small round cell undifferentiated sarcoma is a rare and highly invasive group of malignant bone and soft tissue tumors, often associated with a high misdiagnosis rate. The patient in this case was a 34-year-old male who presented with a two-month history of abdominal pain that worsened over the past two weeks. Elevated levels of tumor markers CA19-9 and CA72-4 were observed. Imaging revealed a substantial, well-vascularized mass in the lower left abdomen, located in the posterior abdominal cavity, invading the descending colon and the root of the small mesentery, and infiltrating the serous layer. The lesion was extensively resected without any postoperative complications. Microscopic examination indicated a combination of mucinous adenocarcinoma (approximately 30%) and small round cell undifferentiated sarcoma (approximately 70%). The patient was followed up for six months, and one month after surgery, a recurrence of the tumor was observed in the left paracolonic sulcus area, with metastases to the abdominal wall, peritoneum, and medial iliac muscles. Chemotherapy and targeted therapy were administered, and the patient currently survives with the presence of tumors. Small round cell undifferentiated sarcoma is an uncommon and highly invasive tumor, and clinical surgeons need to raise their awareness and realize to the maximum extent possible that this disease can be described through a multi-modal combination of immunohistochemistry and genetic test to improve diagnostic accuracy and reduce missed diagnoses. Further research in the field of biology is necessary to explore targeted drugs specifically suitable for this disease.

## 1 Introduction

Small round cell undifferentiated sarcoma is a rare group of malignant bone and soft tissue tumors that primarily affects children and young adults (1, 2). Due to its rarity and lack of characteristic clinical manifestations, it is often misdiagnosed, and it shares similar morphological characteristics with Ewing sarcoma, which is highly invasive. In this report, we present a rare case of a malignant tumor located in the posterior abdominal cavity, involving the descending colon and small mesentery. The pathological diagnosis revealed a case of small round cell undifferentiated sarcoma accompanied by mucinous adenocarcinoma. We discuss its clinicopathological features, molecular genetic characteristics, treatment, and prognosis, aiming to increase clinical surgeons’ awareness of this disease and reduce its misdiagnosis and missed diagnosis rates.

## 2 Case report

**Figure 1:**
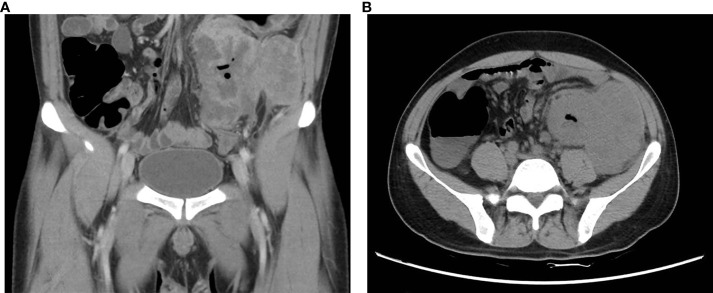
Preoperative CT images of this patient. **(A)** Coronal section CT showed blurred boundaries between the descending colon mass and the adjacent peritoneum and abdominal wall, as well as blurred periintestinal fat spaces (yellow arrow); **(B)** Transverse plane CT revealed irregular thickening of the intestinal wall of the descending colon (yellow arrow), forming soft tissue masses approximately 9.9*8.5*10.4 cm.

A 34-year-old male patient was admitted to the hospital with a history of recurrent abdominal pain lasting for over 2 months. A mass in the left lower abdomen had been present for more than two weeks. Physical examination revealed a palpable mass measuring approximately 6 cm * 8 cm in size in the left lower abdomen. Tumor marker tests showed a carbohydrate antigen CA72-4 level of 37.2 U/ml and a carbohydrate antigen CA19-9 level of 50.7 U/ml. Furthermore, applying a negative enrichment targeted PCR technique, peripheral blood circulating tumor cells (CTCs) were detected in whole blood samples treated with EDTA anticoagulation. The level of CTCs in the peripheral blood was 9.90 FU/3 ml (with a reference value of ≤ 8.70 FU/3 ml). It is worth mentioning that at the point of the initial diagnosis, the patient’s lactate dehydrogenase level was noticeably increased at 448U/L. A B-ultrasound (BUS) examination revealed a significant mass in the left lower abdomen with abundant blood supply. Abdominal computed tomography (CT) indicated a high possibility of descending colon cancer involving the surrounding fat space, adjacent peritoneum, and abdominal wall, with potential peri-intestinal and para-aortic lymph node metastasis (**Figures 1A, B**).

**Figure 2:**
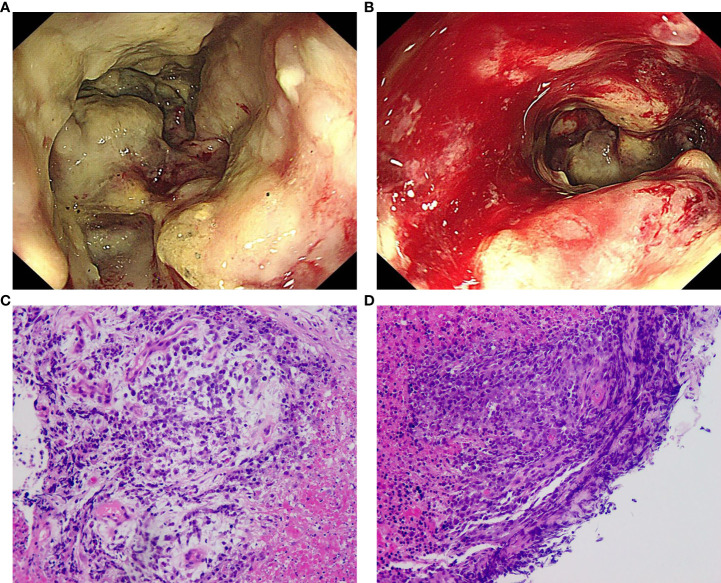
Colonoscopy and pathological results of biopsy. **(A)** The tumor of the descending colon surrounded the whole intestinal cavity, the intestinal cavity was narrow, the surface of the tumor was rough and uneven, and the mucosa was eroded; **(B)** The descending colon mass was easily bled when touched; **(C)** (H&E) The malignant tumor with significant necrosis and interstitial mucinous degeneration; **(D)** (H&E) The tumor cells were small round with sparse cytoplasm, round nuclei and obscure nucleoli, suggesting small round cell undifferentiated sarcoma.

**Figure 3:**
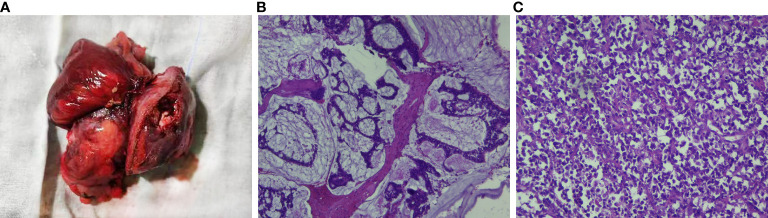
Intraoperative image of this patient and pathological examination results. **(A)** The mass was of the giant type, with a size of 15*13*9 cm. Most of the mass was raised on the serous surface of the intestinal duct, involving the whole intestinal wall to the surrounding fat, with obvious necro-sis and a small amount of bleeding. **(B)** (H&E) The tumor cells were ethmoid and glandular tubes floating in the mucous pool, suggesting mucinous adenocarcinoma. **(C)** (H&E) The tumor cells were diffusely arranged with sparse cytoplasm, round nuclei deeply stained, and obscure nucleoli, and the fission was approximately 26/1.732 square millimeters, suggesting small round cell undifferentiated sarcoma.

Colonoscopy revealed that the tumor had a rough and uneven surface with eroded mucosa. Additionally, the tumor appeared to be attached to the surrounding tissues (**Figures 2A, B**). The pathological results suggested that the descending colon was a small round cell undifferentiated sarcoma with interstitial mucinous degeneration (**Figures 2C, D**). Immunohistochemistry results showed the following: CKpan (weak positive), CK8/18 (negative), CD20 (negative), CD3 (negative), TdT (negative), CD99 (positive), NKX2.2 (negative), CD34 (negative), Synaptophysin (positive), Chromogranin A (negative), CD56 (negative), CD117 (negative), Desmin (negative). Intestinal dilatation and the creation of gas-liquid planes were observed in the patient’s decubitus posture and standing abdominal radiographs. A CT scan revealed some gas-liquid planes in the pelvic cavity, suggesting the potential of incomplete intestinal obstruction. Thus, laparoscopic left hemicolectomy was performed under general anesthesia to remove the tumor located in the posterior abdominal cavity. The tumor had a size of approximately 16 cm * 11 cm * 18 cm (**Figure 3A**). It invaded the descending colon and the root of the small mesentery, extending into the serosal layer. The surgical procedure involved posterior abdominal mass resection, left hemicolectomy, partial small bowel resection, and ureterolysis. Microscopic examination of the tumor revealed that it was composed of two distinct components (**Figure 3B**): the tumor cells were deposited in a cribriform and tubular shape floating in the mucus pool, and another kind of tumor cell was diffusely arranged in sheets. The cells were small round cells with sparse cytoplasm, round and deeply stained nuclei, unclear nucleoli, and mitotic figures of approximately 26 per 1.732 square millimeters (**Figure 3C**).

Based on the pathological examination, the tumor showed patchy tumor necrosis (approximately 30%) and infiltration into the subserosal adipose tissue of the intestinal wall. The small intestine was not involved, and no vascular tumor thrombus or nerve invasion was found. However, tumor tissue was detected at the basal incisal margin in fibrous adipose tissue. The final pathological diagnosis indicated a combination of mucinous adenocarcinoma with small round cell undifferentiated sarcoma. Immunohistochemistry results showed that the small round cell component expressed Vimentin (positive), CD30 (negative), Myeloperoxidase (negative), ALK (negative), WT-1 (scattered weak positive), CD43 (negative), MITF (focal weak positive), MelanA (negative), Myogenin (negative), TFE3 (negative), DOG1 (negative), and a high proliferation rate indicated by Ki-67 (positive, 70%). The mucinous adenocarcinoma component expressed CKpan (positive), CK20 (positive), CK8/18 (positive), and a high proliferation rate indicated by Ki-67 (positive, 90%). Mismatch repair protein screening showed intact expression of MLH1, PMS2, MSH2, and MSH6, suggesting proficient mismatch repair (pMMR). Our pathology results were checked jointly by the reporting doctor, Professor Qin Zhenzhao, and the reviewing doctor, Professor Chen Jun, to ensure the accuracy of the pathology report. They also pointed out that the components of small round cell undifferentiated sarcoma in this case could not be further classified due to limited detection conditions, and it is recommended to send them to domestic specialist consultation or to qualified institutions for genetic testing for a definitive diagnosis. Unfortunately, the patient in this case did not submit for genetic testing due to economic reason.

After extensive resection without complications, a one-month postoperative CT reexamination revealed a soft tissue density mass shadow in the left paracolic sulci, showing uneven enhancement. Tumor recurrence was suspected, involving the abdominal wall, peritoneum, and iliac medial muscle group, suggesting metastasis. The patient received three cycles of VAC chemotherapy (vincristine, doxorubicin, and cyclophosphamide), resulting in a reduction in the size of the lesions on CT examination. Following further evaluation and exclusion of contraindications, the patient underwent targeted therapy with Anlotinib. As of August 2023, the patient is alive with tumor and continues to be monitored. Long-term follow-up is necessary for proper management of the condition.

## 3 Discussion

The diagnosis and treatment process of this patient has been reviewed. Prior to the operation, the patient’s clinical manifestations, colonoscopy, and CT results indicated that the lesion originated from the colon. Furthermore, we did not initially treat the patient with neoadjuvant chemotherapy or radiotherapy as recommended in the National Comprehensive Cancer Network (NCCN) clinical Practice Guidelines for soft tissue sarcoma due to concern that the patient would develop intestinal obstruction (3). However, during the operation, it was discovered that the primary lesion actually originated from the posterior abdominal cavity. It was clear that the lesion in the colon was caused by the invasion of a giant sarcoma in the posterior abdominal cavity. As a result, a giant small round cell undifferentiated sarcoma in the posterior abdominal cavity poses challenges in clinical diagnosis due to their highly invasive nature, lack of specific manifestations, and high misdiagnosis rate. The final diagnosis relies on surgical resection and pathological examination. Currently, research on the molecular mechanism and treatment focus of colon malignant tumors has reached a relatively mature stage (4). This includes exploring microsatellite instability, CpG island hypermethylation, and various gene mutations associated with carcinogenesis (5, 6). However, the correlation between small round cell undifferentiated sarcoma and malignant colon tumors remains unclear, and further investigation is needed to explore any related molecular mechanisms.

Small round cell undifferentiated sarcoma is a group of malignant tumors affecting bone and soft tissues (7). In the 5th edition of the WHO classification of soft tissue and bone tumors, undifferentiated round cell sarcoma is divided into several types, including Ewing’s sarcoma, CIC rearrangement sarcoma, BCOR rearrangement sarcoma, sarcoma with EWSR1-non-ETS fusion, and adhesive small round cell tumor (8). The most common types of small round cell undifferentiated sarcoma are CIC rearrangement sarcoma (CIC-DUX4, CIC-DUX4L, CIC-FOXO4) and BCOR rearrangement sarcoma (BCOR-CCNB3, BCOR-MAML3, ZC3H7B-BCOR) (9), and the former is driven by a fusion between the tumor suppressor Capicua (CIC) and DUX4 (10). DUX4 is the most common translocation in CIC rearrangement sarcoma, often resulting from t (4, 11) (q53; q13) or t (10, 11) (q26; q13) (12, 13). Literature reports on small round cell undifferentiated sarcoma, whether domestic or international, are mostly limited to relatively small retrospective cases. Some cases of small round cell undifferentiated sarcoma have been reported in the lung (14), bone (15), and deep abdominal wall (16), but cases of colonic malignant tumors associated with small round cell undifferentiated sarcoma are rare. Only one case of circular cell CIC rearrangement sarcoma in the colon was reported in 2017 by Maghrebi et al. Therefore, in our patient, small round cell undifferentiated sarcoma accompanied by mucinous adenocarcinoma of the colon is a rare occurrence in clinical practice, because it was originally thought to be a colon cancer. The clinical manifestations and related auxiliary examinations are nonspecific. Pathology and immunohistochemistry play a crucial role in diagnosis due to their high sensitivity and specificity (17, 18). Further genetic testing is necessary to determine the tumor type (19). In addition, if the gene fusion associated with CIC is not detected, it is difficult to distinguish it from Ewing sarcoma (11). However, in this particular case of small cell undifferentiated sarcoma, further classification was not possible due to limitations in our hospital’s resources. Therefore, clinicians must be aware that diagnosing this disease requires a combination of symptomatology, radiology, histopathology, and immunohistochemistry (20). The widespread use of morphology, immunohistochemistry, and advanced molecular techniques is crucial in distinguishing various types of small round cell undifferentiated sarcoma, as different tumor types exhibit distinct molecular characteristics, clinical histories, and treatment sensitivities. A comprehensive molecular evaluation should be conducted when a diagnosis is suspected to facilitate molecular diagnosis and staging, ultimately leading to more effective treatment and prognosis (21–23).

Small round cell undifferentiated sarcoma is a highly invasive malignant tumor that shares morphological similarities and partially overlaps with other types of small round cell tumors in immunohistochemical detection (7, 24). It is extremely rare in clinical practice, with few reports in the literature, a high misdiagnosis rate, and poor prognosis (18). Interestingly, recent reports have mainly focused on cases of Ewing sarcoma of the colon/PNET combined with liver metastasis, with an overall cure rate of only 20% for metastatic Ewing sarcoma (25, 26). It is noteworthy that the median survival time for small round cell undifferentiated sarcoma is less than 2 years (19, 27), and the 5-year survival rate for 57 patients with CIC rearrangement sarcoma was 43%, lower than that of Ewing sarcoma (28). Furthermore, a study involving 148 patients with small round cell undifferentiated sarcoma (60% CIC rearrangement sarcoma, 22% BCOR-CCNB3 rearrangement sarcoma, 18% unclassified small round cell undifferentiated sarcoma) reported a 3-year overall survival rate of 92.2% for BCOR-CCNB3 patients, 39.6% for CIC rearrangement sarcoma patients, and 78.7% for unclassified small round cell undifferentiated sarcoma patients after a median follow-up of 42.7 months (29). Notably, as mentioned above, the patient in this case had a significant elevation of lactate dehydrogenase upon admission, which suggested a potential recurrence only one month after surgery. This is consistent with literature reporting that elevated lactate dehydrogenase at first diagnosis and initial recurrence in patients with Ewing sarcoma signal a poor prognosis (30). Therefore, a more proactive approach to the treatment of this rare disease is required. Unfortunately, there are currently no standardized guidelines for its diagnosis and treatment both domestically and internationally (24). Small round cell undifferentiated sarcoma is morphologically similar to Ewing’s sarcoma, so the treatment is often modeled after Ewing’s sarcoma (29). Current treatment strategies are primarily based on those used for other types of small round cell malignant tumors, including surgical resection, chemotherapy, and radiotherapy (19, 27, 31). However, small round cell undifferentiated sarcoma tends to exhibit resistance to chemotherapy (27, 32). Although studies have identified CIC translocation as a driving factor for tumor growth and metastasis in CIC rearrangement sarcoma, targeted therapies specifically for this condition are not yet available (33). Nevertheless, some studies have shown promising results with targeted drugs such as Plitidepsin, which targets the translation factor eEF1A1 in similar soft tissue sarcoma cases (33). CDK 4/6 inhibitor palbociclib and soft tissue sarcoma drug trabectedin have been found to inhibit the growth of CIC-DUX4 sarcoma in mice (11), and molecular targeted drugs like Crizotinib can partially control the growth of CIC rearrangement sarcoma (34). Besides, based on the ALTER0203 study, a Phase 2b randomized, double-blind, placebo-controlled multicenter clinical study of Anlotinib in the treatment of advanced soft tissue sarcoma, Anlotinib was approved by the National Medical Products Administration (NMPA) for second-line treatment of soft tissue sarcoma in June 2019. Also based on this study, the Chinese Society of Clinical Oncology (CSCO) Sarcoma Expert Group upgraded the level of recommendation and evidence level of Anlotinib to Level I recommendation and Level 1A evidence, respectively (35). In this case report, the patient received targeted therapy after postoperative radiotherapy, resulting in reduced lesions upon reexamination. Therefore, targeted therapy for small round cell undifferentiated sarcoma may provide new hope for survival. Further studies are needed to determine targeted therapies for each subtype of small round cell undifferentiated sarcoma.

## 4 Conclusion

In conclusion, small round cell undifferentiated sarcoma is an extremely rare form of sarcoma, and a highly invasive tumor with nonspecific clinical manifestations and a high rate of misdiagnosis. The diagnosis primarily relies on pathological and immunohistochemical examination. Treatment typically involves a combination of surgery, radiotherapy, and chemotherapy. However, small round cell undifferentiated sarcoma exhibits high resistance to chemotherapy and carries a poor prognosis. Therefore, further research is needed to enhance the diagnostic accuracy of this condition. Additionally, molecular diagnosis and staging hold promising prognostic value, and more biological studies are necessary to explore targeted therapeutic approaches for this disease.

## Data availability statement

The raw data supporting the conclusions of this article will be made available by the authors, without undue reservation.

## Ethics statement

The studies involving humans were approved by Guangxi Medical University Cancer Hospital. The studies were conducted in accordance with the local legislation and institutional requirements. The participants provided their written informed consent to participate in this study. Written informed consent was obtained from the individual(s) for the publication of any potentially identifiable images or data included in this article.

## Author contributions

YW, ZZ, CL, XH, WT, XM and JL conceived the idea for this article. YW drafted the manuscript. XM and JL proposed constructive revisions to the original manuscript. JL approved the final version of the manuscript. All authors contributed to the article and approved the submitted version.
